# Antimicrobial resistance patterns of WHO priority pathogens isolated in hospitalized patients in Japan: A tertiary center observational study

**DOI:** 10.1371/journal.pone.0294229

**Published:** 2024-01-11

**Authors:** Tania Tabassum Nisa, Daisaku Nakatani, Fumie Kaneko, Toshihiro Takeda, Ken Nakata

**Affiliations:** 1 Department of Global and Innovative Medicine, Osaka University Graduate School of Medicine, Yamadaoka, Suita, Osaka, Japan; 2 Department of Medical Informatics, Osaka University Graduate School of Medicine, Yamadaoka, Suita, Osaka, Japan; Carol Davila University of Medicine and Pharmacy, ROMANIA

## Abstract

**Background:**

After issuing the “Global action plan on antimicrobial resistance” in 2015, the World Health Organization (WHO) established a priority pathogens list for supporting research and development of novel antimicrobials. We conducted a comprehensive analysis of the WHO priority organisms in a Japanese tertiary hospital to apprehend the local AMR epidemiology.

**Methods:**

Data were obtained from electrical medical records in Osaka University Hospital between January 2010 and March 2021. The critical, high, and medium “priority pathogens list” categories of the WHO were used to compare results between the early (2010–2015) and late (2016–2021) phases.

**Results:**

Out of 52,130 culture-positive specimens, a total of 9,872 (18.9%) contained WHO priority isolates. In comparison to early phases, late phases were likely to have higher rates of carbapenem resistance in *Pseudomonas aeruginosa* (15.7% vs 25.0%, P<0.001), 3^rd^ generation cephalosporin resistance in *Escherichia coli* (11.5% vs 17.8%, P<0.001) as well as *Klebsiella pneumoniae* (1.6% vs 4.4%, P<0.001), and ampicillin resistance in *Haemophilus influenzae* (2.4% vs 3.9%, P<0.001). After 2015, however, the proportion of methicillin-resistant and vancomycin-intermediate *Staphylococcus aureus* was low. In this study, in-hospital mortality was comparable among patients with resistance to the three WHO priority pathogen types: critical (5.9%), high (3.9%), and medium (3.8%), and no significant change was observed between two phases in each category. However, significant interactions for in-hospital mortality were observed in subgroup analyses between “critical priority” AMR and the presence of comorbid conditions, such as chronic kidney disease or diabetes mellitus.

**Conclusions:**

To implement better antimicrobial stewardship policies and practices, local priority pathogens and “high-risk” patients for in-hospital death need to be acknowledged and evaluated periodically.

## Introduction

The discovery of penicillin and the subsequent introduction of other new antibiotics in the following decades drastically reduced global mortality [1]. These days, regrettably, many important human and animal pathogens have developed antimicrobial resistance (AMR) for a variety of reasons, including misuse and overuse of antimicrobials [2]. Treating infectious diseases has become more complicated because of these resistances, which are leading to a prolonged illness, disability, and death [2,3]. By 2050, there would be 10 million deaths per year, and 24 million people may fall into extreme poverty due to AMR [4–6]. Considering the catastrophe, in 2015, the World Health Assembly adopted a Global Action Plan on AMR [7]. To support the policy and further prioritize research and development of new antimicrobials, the World Health Organization (WHO) published a priority pathogens list (PPL) of twelve bacterial families and stratified them as critical, high, and medium priorities [8]. The WHO-PPL was issued based on data from several sources regarding global mortality, the availability of effective therapy, and the health-care burden due to AMR [8–10].

The Japanese government published a national action plan on AMR in April 2016 intending to implement One Health surveillance regarding AMR bacteria isolated from humans, animals, food, and the environment [11]. Since 2017, the AMR One Health Surveillance Committee has been publishing Nippon AMR One Health Reports (NAOR) for identifying the status and issues related to AMR inside Japan [12–15]. Although numerous reports narrated AMR in Japanese individuals, to our best knowledge no records were published based on WHO-PPL. Yet, generating AMR-related evidence from a global priority standpoint is critical not only for the quick obtainment of global policies but also to recognize and promptly respond to a probable local AMR trend. Based on WHO priority pathogen lists [8], we, therefore, aimed to present a comprehensive analysis of the prevalence and outcomes of AMR among organisms isolated from hospitalized patients at Osaka University Hospital. This hospital is a well-recognized tertiary referral center in Japan with 1,086 beds serving mainly immunocompromised and terminally ill patients with previous difficult and unsuccessful treatment histories. In our study, we have compared AMR epidemiology before and after the “Global Action Plan” [7] endorsement to understand the local priorities that can be applied to revise stewardship in this tertiary healthcare center or elsewhere.

## Methods

### Study design and endpoints

This study was performed using the electronic medical records (EMRs) of the Osaka University Hospital database. We retrospectively retrieved patients’ EMRs who had been hospitalized between January 1, 2010, and March 31, 2021. To maintain confidentiality, we anonymized all personal information and delinked it from EMR numbers before analysis. The institutional review board at Osaka University decided to waive the requirement that participants provide informed consent. Therefore, we did not obtain informed consent from our study participants. Instead, an “opt-out” strategy was used [16] based on the regulations of the same review board. All hospitalized patients who underwent bacterial culture and antimicrobial susceptibility testing (AST), irrespective of departments or wards, were included in our research. We had to reckon on the attending physicians’ judgements for specimen selection as the samples were not pre-set due to the retrospective nature of this study. The Laboratory for Clinical Investigation, at Osaka University Hospital, performed all the bacteriological tests and reported the results to the physicians based on Clinical and Laboratory Standards Institute (CLSI) guidelines [17]. For pathogen identification and AST, the MicroScan WalkAway plus System and/or Microflex LT/SH by Bruker Daltonics were used. In the electronic records, the AST results marked as “not found” were excluded from this research. To avoid duplication, the reports containing repeated samples from the same specimen site of the same subject were excluded from the analyses. However, records with different specimen sources of the same subject were included. Pathogens and AST other than those referring to the WHO priority pathogen list (PPL) were excluded from the analysis. The WHO-PPL was used to define AMR as critical, high, or medium categories [8,10], and in-hospital mortality was regarded as the outcome. To compare the results, the total study period was divided into early (2010–2015) and late (2016–2021) phases. The study was approved by the institutional review board at Osaka University (Approval number: 21223) before accessing the EMRs and followed the guidelines of the Declaration of Helsinki as revised in 2013.

### Statistical analysis

Continuous variables were reported as means with standard deviation (SD) or medians (IQR) and the student’s t-tests were used to compare them. Categorical variables were summarized with counts and percentages and were compared with Chi-square tests. Confidence intervals (CI) for percentages were based on the exact binomial distribution. Multivariate logistic regression analyses were performed to identify the risk factors for in-hospital mortality in “critical-priority” AMR patients with different demographics. Variables inserted into the regression model included age, sex, departments, chronic kidney disease, and diabetes mellitus that were considered clinically important. All the statistical tests were 2-tailed, and p < 0.05 or p for interaction < 0.1 was considered significant. All analyses were performed using IBM SPSS Statistics 27.0.1 (Chicago, IL, USA).

## Results

During the study period, a total of 52,130 specimens showed culture positivity, of which 9,872 (18.9%) contained WHO-PPL [8] pathogens. The highest number of WHO-PPL organisms were found in sputum (2,089/9,872), followed by nasopharyngeal swabs (2,015/9,872) and blood (1,156/9,872). A “high Priority” organism, *Staphylococcus aureus*, was the most commonly found (58.8%) WHO-PPL isolate. The other organisms showing high prevalence were *Pseudomonas* aeruginosa (19.0%) and *Escherichia coli* (13.7%), both of which belong to the “critical priority” group **(Table 1)**.

**Table 1:** World Health Organization priority pathogen list (WHO PPL) isolates collected from the Osaka University Hospital between January 1, 2010, and March 31, 2021.

Specimen	Blood	Sterile body fluids	Other fluids	Urine	Stool	Sputum	NP swabs	Other swabs	Tissue	Fluids in the drain tube	Wound	Pus	GIT content	Equipment	Total
**Critical priority isolates**															
*Acinetobacter baumannii*	3 (0.3)	0(0)	0(0)	2 (0.2)	0(0)	6 (0.3)	1(0)	0(0)	1 (0.2)	2 (0.8)	0(0)	0(0)	0(0)	1 (0.2)	16(0.2)
*Pseudomonas aeruginosa*	144 (12.5)	93 (31.3)	19 (10.2)	139 (14.5)	18 (8.5)	635 (30.4)	287 (14.2)	2 (2.9)	86 (17.5)	118 (45.7)	185 (16.6)	45 (9.8)	0(0)	106 (18.9)	1,877(19.0)
***Enterobacteriaceae***															
*Klebsiella pneumoniae*	34 (2.9)	18 (6.1)	3 (1.6)	59 (6.2)	8 (3.8)	25 (1.2)	27 (1.3)	0(0)	5 (1.0)	15 (5.8)	32 (2.9)	6 (1.3)	1 (20.0)	21 (3.8)	254(2.6)
*Escherichia coli*	245 (21.2)	66 (22.2)	13 (7.0)	495 (51.7)	21 (9.9)	101 (4.8)	94 (4.7)	5 (7.1)	37 (7.5)	30 (11.6)	69 (6.2)	47 (10.2)	0 (0)	134 (23.9)	1,357(13.7)
*Serratia spp*.	0(0)	0(0)	0(0)	1(0.1)	0(0)	0(0)	3(0.1)	0(0)	1(0.2)	0(0)	0(0)	0(0)	0(0)	0(0)	5(0.1)
*Proteus spp*.	0(0)	0(0)	0(0)	0(0)	0(0)	0(0)	0(0)	0(0)	0(0)	0(0)	1(0.1)	1(0.2)	0(0)	0(0)	2(0)
*Providencia spp*.	1(0.1)	0(0)	0(0)	0(0)	0(0)	0(0)	0(0)	0(0)	0(0)	0(0)	0(0)	0(0)	0(0)	0(0)	1(0)
**High priority isolates**															
*Staphylococcus aureus*	683 (59.1)	115 (38.7)	140 (74.9)	251 (26.2)	153 (72.2)	1,030 (49.3)	1,479 (73.4)	52 (74.3)	326 (66.3)	92 (35.7)	826 (74.1)	358 (77.8)	4 (80.0)	293 (52.3)	5,802(58.8)
*Enterococcus faecium*	0(0)	0(0)	0(0)	0(0)	6(2.8)	0(0)	0(0)	0(0)	0(0)	0(0)	0(0)	2(0.4)	0(0)	0(0)	8(0.1)
*Helicobacter pylori*	0(0)	0(0)	0(0)	0(0)	0(0)	0(0)	0(0)	0(0)	3(0.6)	0(0)	0(0)	0(0)	0(0)	0(0)	3(0)
*Campylobacter*	10 (0.9)	0(0)	0(0)	0(0)	5(2.4)	0(0)	0(0)	0(0)	0(0)	0(0)	0(0)	0(0)	0(0)	0(0)	15(0.2)
*Neisseria gonorrhoeae*	0(0)	0(0)	0(0)	0(0)	0(0)	0(0)	0(0)	2(2.9)	0(0)	0(0)	0(0)	0(0)	0(0)	0(0)	2(0)
**Medium priority isolates**															
*Haemophilus influenzae*	9(0.8)	0(0)	2(1.1)	5(0.5)	1(0.5)	190(9.1)	59(2.9)	0(0)	14(2.8)	0(0)	1(0.1)	1(0.2)	0(0)	3(0.5)	285(2.9)
*Streptococcus pneumoniae*	27 (2.3)	5(1.7)	10(5.3)	5(0.5)	0(0)	102(4.9)	65(3.2)	9(12.9)	19(3.9)	1(0.4)	0(0)	0(0)	0(0)	2(0.4)	245(2.5)
Total isolates	1,156	297	187	957	212	2,089	2,015	70	492	258	1,114	460	5	560	9,872

NP, nasopharyngeal; GIT, gastrointestinal.

**Fig 1:**
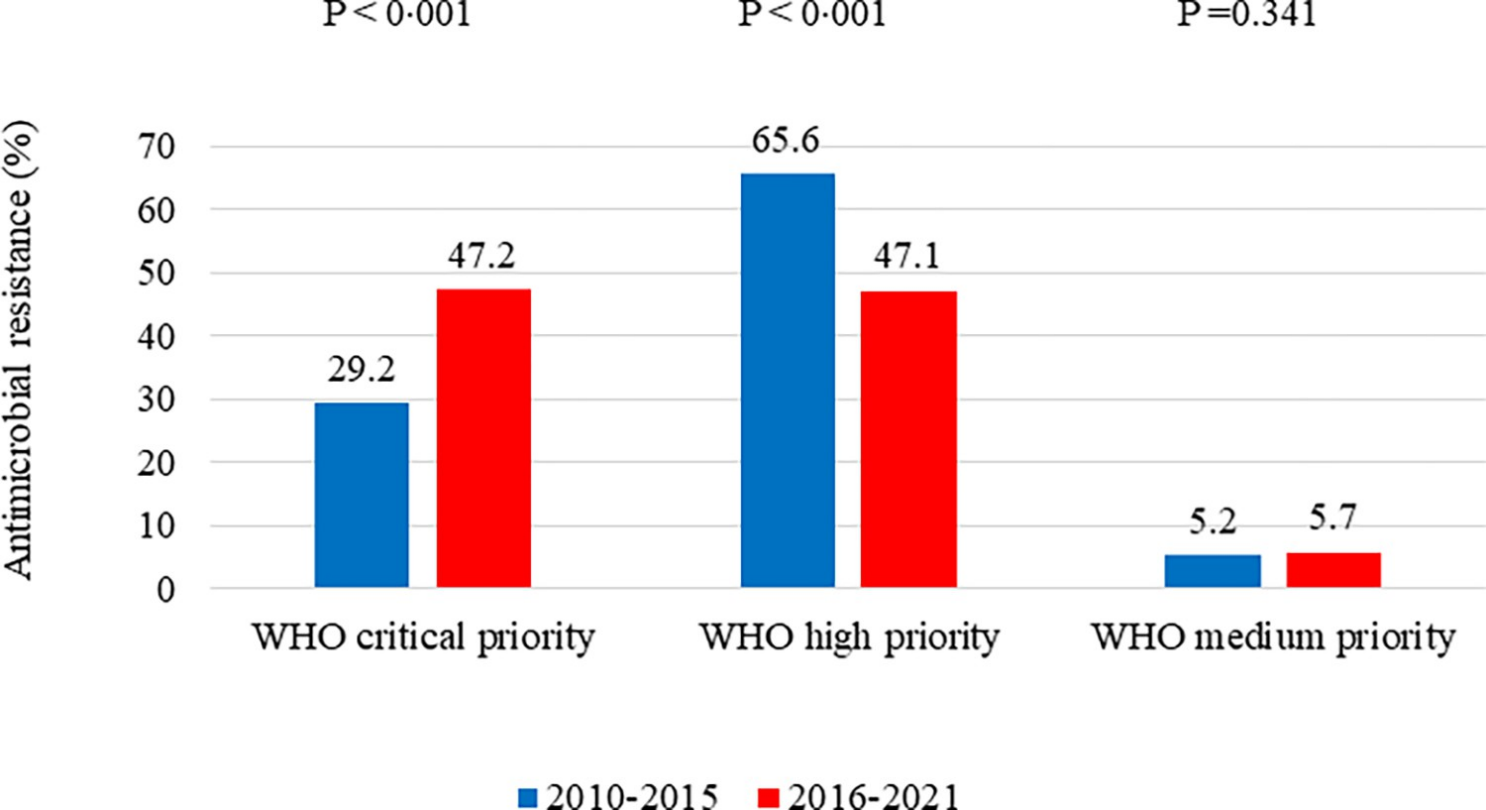
The percentage of antimicrobial resistance (AMR) in World Health Organization Priority Pathogen List (WHO PPL) isolates from the Osaka University Hospital between January 1, 2010, and March 31, 2021.

In the late (2016–2021) phase, the percentage of resistance in “critical priority” pathogens was significantly higher (29.2% vs 47.2%), while the proportion was lower for the “high priority” isolates (65.6% vs 47.1%). However, no significant difference in the AMR% of "medium priority" organisms was observed between the two phases **(Fig 1)**.

Among the “critical priority” organisms, a significantly higher proportion of resistance to carbapenem groups of antimicrobials was observed in *Pseudomonas aeruginosa* during the 2016–2021 phase. Additionally, there was an increase in resistance to 3^rd^ generation cephalosporins found in some *Enterobacteriaceae* belonging to the ‘critical priority’ group, particularly *Escherichia coli* and *Klebsiella pneumoniae*, after 2015. However, in the “High priority” group, the proportion of methicillin-resistant and vancomycin-intermediate *Staphylococcus aureus* was lower between 2016 and 2021. *Haemophilus influenzae*, a “medium priority” pathogen, demonstrated higher ampicillin resistance in the late phase **(Table 2)**.

**Table 2:** Percentage of resistance in World Health Organization priority pathogen list (WHO PPL) organisms to specific antimicrobials, collected from the Osaka University Hospital between January 1, 2010, and March 31, 2021.

	Pathogen	2010–2015	2016–2021	All	P-Value
		N = 6,374	N = 3,498	N = 9,872	
Critical priority	*Acinetobacter baumannii*, CR	16(0.3)	0(0)	16(0.2)	0.003
	*Pseudomonas aeruginosa*, CR	1,003(15.7)	874(25.0)	1,877(19.0)	<0.001
	***Enterobacteriaceae***				
	*Klebsiella pneumoniae*, 3GCR	101(1.6)	153(4.4)	254(2.6)	<0.001
	*Escherichia coli*, 3GCR	735(11.5)	622(17.8)	1,357(13.7)	<0.001
	*Serratia spp*., 3GCR	5(0.1)	0(0)	5(0.1)	0.098
	*Proteus spp*., 3GCR	0(0)	2(0.1)	2(0)	0.056
	*Providencia spp*., 3GCR	1(0)	0(0)	1(0)	0.459
High priority	*Staphylococcus aureus*, *M*R	3,890(61.0)	1,611(46.1)	5,501(55.7)	0.000
	*Enterococcus faecium*, VR	6(0.1)	2(0.1)	8(0.1)	0.537
	*Staphylococcus aureus*, VI	74(1.2)	2(0.1)	76(0.8)	<0.001
	*Staphylococcus aureus*, MR, VI	203(3.2)	22(0.6)	225(2.3)	<0.001
	*Helicobacter pylori*, ClaR	0(0)	3(0.1)	3(0)	0.019
	*Campylobacter*, FQR	8(0.1)	7(0.2)	15(0.2)	0.363
	*Neisseria gonorrhoeae*, FQR	0(0)	2(0.1)	2(0)	0.056
Medium priority	*Streptococcus pneumoniae*, PNS	182(2.9)	63(1.8)	245(2.5)	0.001
	*Haemophilus influenzae*, AmpR	150(2.4)	135(3.9)	285(2.9)	<0.001

CR, carbapenem-resistant; 3GCR, 3rd generation cephalosporin-resistant; MR, methicillin-resistant; VR, vancomycin-resistant; VI, vancomycin-intermediate; claR, clarithromycin-resistant; FQR, fluoroquinolone-resistant; PNS, penicillin-non-susceptible; AmpR, ampicillin-resistant.

The cultures with the WHO priority organisms belonged to 1,601 patients. Of them, 969 (60.5%) were male subjects. Compared to the early phase, the frequency of chronic diseases such as hypertension, chronic kidney disease, diabetes, and cancer was lower in the late phase **(S1 Table)**. However, there was no significant difference in in-hospital mortality between the two phases in patients with WHO-PPL resistance. **(S2 Table)**.

**Fig 2:**
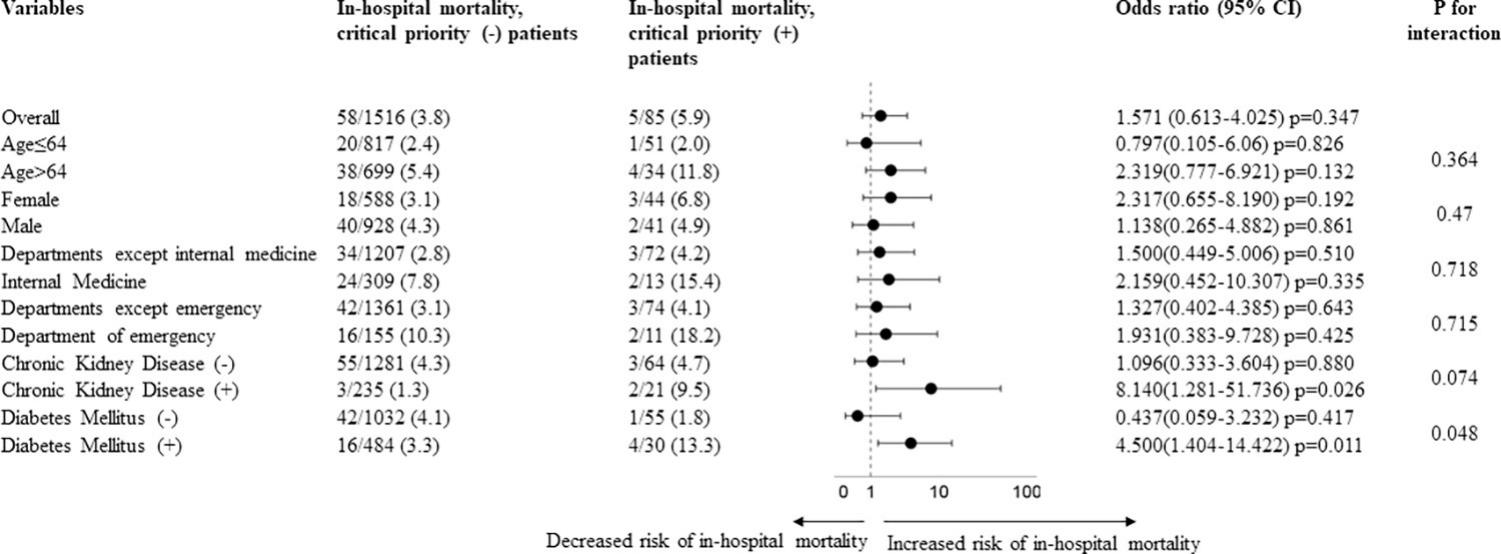
Relationship between WHO “critical priority” resistance and in-hospital mortality in various clinical profiles.

Considering the clinical importance of “critical priority” resistance and the higher prevalence after 2015, other factors associated with this resistance and negative outcomes were further examined. Among non-chronic kidney disease (CKD) patients, there was no significant difference in in-hospital mortality between patients with and without "critical priority" AMR (4.7% vs 4.3%), while in patients with CKD (+), in-hospital mortality was significantly higher in "critical priority" AMR (+) than those without it (9.5% vs 1.3%) with an odds ratio of 8.140 (95% CI; 1.281–51.73). A significant interaction of 0.074 was observed for in-hospital mortality between AMR and the presence of CKD. Additionally, among patients with no diabetes mellitus (DM), there was a trend toward lower in-hospital mortality in "critical priority" AMR (+) compared to "critical priority" AMR (-) (1.8% vs 4.1%). Whereas among diabetic patients, a significantly higher risk for in-hospital mortality was noticed in "critical priority" AMR (+) than those without it (13.3% vs 3.3%) with an odds ratio of 4.500 (95% CI; 1.404–14.422). There was also a significant interaction of 0.048 for in-hospital mortality between "critical priority" AMR and the presence of DM **(Fig 2).**

To assess the antimicrobial usage in Osaka University Hospital, we considered the antibiotics mentioned in the WHO-PPL. Our data reveals a substantial increase in carbapenem usage among hospitalized patients from 2016 to 2021. Additionally, a rise in the usage of antifungals, particularly as adjunctive/combination therapy alongside other antibiotics, was observed during the late phase. In contrast, a lower proportion of usage was observed for 3rd generation cephalosporins during the same late phase **(Table 3)**.

**Table 3:** Antimicrobial use in Osaka University Hospital wards from January 1, 2010, to March 31, 2021.

	2010–2015	2016–2021	All	P-value
	N = 41,772	N = 25,682	N = 67,454	
Carbapenems usage (%)	2,838(6.8)	3,056(11.9)	5,894(8.7)	<0.001
3GC usage (%)	4,978(11.9)	1,584(6.2)	65,62(9.7)	<0.001
Antifungals usage (%)	4,546(10.9)	3,090(12.0)	7,636(11.3)	<0.001
Other antimicrobials usage (%)	2,9410(70.4)	1,7952(69.9)	47,362(70.2)	0.164

3GC, 3rd generation cephalosporins.

## Discussion

A 10-year trend in global resistance was considered one of the MCDA (multi-criteria decision analysis) benchmarks during the categorization of the WHO-PPL organisms in 2017 [9]. The pattern showed that the prevalence of third-generation cephalosporin-resistance in *Klebsiella pneumoniae* and *Escherichia coli* ranged from 16–30% in Japan, while carbapenem resistance in *Acinetobacter baumannii* and *Pseudomonas aeruginosa* ranged from 31–50%. It was anticipated that resistance of these organisms to antibiotics would continue to rise in the Western Pacific WHO area, including Japan [9]. Most of our current study findings support this WHO report. The higher proportion of resistance in the “critical priority” organisms even after the announcement of the 2015 global action plan is extremely important and should be regarded as a serious health threat. The only exception was observed for the *Acinetobacter baumannii* from the “critical priority” group, demonstrating 0% carbapenem resistance in the late phase in our hospital.

Another article, the Nippon AMR One Health Report (NAOR-2020) demonstrated that, in 2019, the proportion of resistance in *P*. *aeruginosa* to imipenem was 16.2% and meropenem was 10.6% in Japanese patients [15]. These results are non-comparable to our current study, as we detected 15.7% carbapenem resistance in *Pseudomonas* before 2015 and that further increased to 25% in the 2016–2021 period. However, comparisons are difficult as the distribution of AMR depends on the category of hospitals or the type and size of the samples. NAOR obtained data from Japan Nosocomial Infections Surveillance (JANIS) funded by the Ministry of Health, Labour and Welfare, Japan [18].

*P*. *aeruginosa* and *Enterobacteriaceae* are Gram-negative organisms and are regarded as very high healthcare burdens with limited treatment options. *P*. *aeruginosa* is considered an opportunistic pathogen causing healthcare-associated infections (HAIs), from which immunocompromised patients mostly suffer [19,20]. Based on a German study targeting six tertiary centers, 9.5% of the hospitalized patients were carriers of 3rd generation cephalosporin resistant *Enterobacteriaceae*, another HAI pathogen [21]. Our study clearly documented that more patients were admitted into both the major and minor surgical departments in Osaka university hospital (OUH) in the late phase. As a tertiary referral hospital, the surgery departments often deal with critically ill patients with advanced-stage malignancies, ongoing chemotherapies, organ transplants, or requiring complicated operations in OUH. These situations might cause long-term hospital stay for the patients and increase the chance of HAIs with resistant pathogens in the late phase.

The resistance proportion of the “high” category, *Staphylococcus aureus* to methicillin (MRSA), which once presented a higher prevalence in Japan (51%) than in most countries [11], showed a significantly lower proportion (46.1%) after 2015 in our study. This improvement can be credited to the recent promotion of antibiogram utilization for MRSA at our hospital, specifically in complex cases or patients with mixed infections. Furthermore, the decrease in MRSA bloodstream infections at Osaka University Hospital can also be attributed to a decline in strains associated with healthcare exposure risk. These positive outcomes were achieved through the implementation of enhanced antibiotic stewardship initiatives and the engagement of an infectious control team. Notably, NAOR-2020 is also consistent with our results, representing a proportion of 47.4% MRSA in patients [15].

From the “medium priority” group, the WHO-PPL report speculated a downward trend of penicillin non-susceptibility in *S*. *pneumoniae* but an upward trend of *Haemophilus influenzae* to ampicillin in the western pacific region [9]. Our results comply with the WHO hypothesis and demonstrate a similar pattern in the late phase.

Mortality was one of the key qualitative variables in the MCDA model used by WHO to classify different bacteria into three separate categories [9]. Yet, in-hospital death was comparable among patients with critical (5.9%), high (3.9%), and medium (3.8%) priority resistance in this study. Although the frequency of in-hospital mortality was more than doubled with "critical priority" resistance in the late phase, the results could not reach statistical significance due to small sample sizes and might not be appropriate to compare with other studies.

However, in subgroup analyses, significant correlations between "critical priority" AMR and the presence of comorbid diseases, such as chronic kidney disease (CKD) and diabetes mellitus, were observed for in-hospital mortality. Usually, end-stage renal disease is linked to a variety of changes in the immune system and both anti-inflammatory interleukin (IL-10) and proinflammatory cytokines (TNF-, IL-6) are elevated [22]. On the other hand, long-term hyperglycaemic exposure is a common feature of diabetes mellitus, which also impairs immunity. Diabetes can induce additional concomitant disorders such as vasculopathy, neuropathy, etc. Multiple previous studies have shown that subjects with impaired renal function or diabetes mellitus are more likely to develop multidrug-resistant infections [23,24]. Therefore, patients presented with these coexisting conditions and resistant “critical priority” infections had higher risks of mortality.

Kidney dysfunction or diabetes at admission may be an indicator of the need for closer attention to microbial culture results, requiring a subsequent change in treatment strategy to ensure an improved prognosis.

Several limitations of our study warrant mention. To begin with, there is no guarantee that the quality controls in our retrospective data will meet the standards of a well-conducted prospective study. Second, the findings of this tertiary center may not be representative of AMR distributions in primary and community care hospitals. Third, we could not rule out selection bias because the bacterial cultures and AST were chosen by the attending physicians. Fourth, the quality of all microbial tests is hard to ensure as they were performed by different people throughout the study period. The medical reports were manually entered into the electronic system, which is also prone to human error. Furthermore, we were unable to obtain information on the patients’ antimicrobial dosage and duration.

## Conclusions

The antimicrobial resistance pattern was indifferent from the prediction of the World Health Organization during the 12 years period at Osaka University Hospital. The analysis also revealed that patients with CKD or diabetes mellitus and WHO “critical priority” AMR infection have a higher likelihood of in-hospital mortality at least in tertiary care centers in Japan. This information may increase the awareness among physicians about the prognostic value of rapid “critical priority” AMR infection tests at admission and aid in the choice of initial antibiotics. Additionally, the disparity in AMR patterns between this study and national Japanese reports provides compelling evidence that AMR findings should be documented and locally evaluated periodically to steer regional priorities and review antimicrobial stewardship programs.

## Supporting information

S1 Table On admission profiles of patients with antimicrobial resistance (AMR) based on World Health Organization priority pathogen list (WHO-PPL). (PDF)

S2 Table In-hospital mortality in patients with antimicrobial resistance (AMR) based on World Health Organization priority pathogen list (WHO PPL). (PDF)

## Abbreviations

Abbreviation: Definition
WHO: World Health Organization
AMR: Antimicrobial Resistance
PPL: Priority Pathogen List
EMRs: Electronic Medical Records
AST: Antimicrobial Susceptibility Testing
CLSI: Clinical And Laboratory Standards Institute
SD: Standard Deviation
CI: Confidence Intervals
CKD: Chronic Kidney Disease
NP: Nasopharyngeal
GIT: Gastrointestinal
CR: Carbapenem-Resistant
3GCR: 3rd Generation Cephalosporin-Resistant
MR: Methicillin-Resistant
VR: Vancomycin-Resistant
VI: Vancomycin-Intermediate
claR: Clarithromycin-Resistant
FQR: Fluoroquinolone-Resistant
PNS: Penicillin-Non-Susceptible
AmpR: Ampicillin Resistant
BMI: Body Mass Index
WBC: White Blood Cells
RBC: Red Blood Cells
BUN: Blood Urea Nitrogen
eGFR: Estimated Glomerular Filtration Rate
ASAT: Aspartate Aminotransferase
ALT: Alanine Transaminase
γGTP: Gamma-Glutamyl Transferase
OUH: Osaka University Hospital
