# Supplementary material for: Antimicrobial resistance patterns of WHO priority pathogens isolated in hospitalized patients in Japan: A tertiary center observational study
Source: PLoS One. 2024 Jan 11;19(1):e0294229. doi: 10.1371/journal.pone.0294229 (PMC10783704; doi:10.1371/journal.pone.0294229)
Supplement: S1 Table — (PDF) [file pone.0294229.s001.pdf]

On admission profiles of patients with antimicrobial resistance (AMR) based on World Health Organization priority pathogen list (WHO-PPL)

	2010-2015	2016-2021	all	P- value
	N=963	N=638	N=1,601	
Male, n (%)	573(59.5)	396(62.1)	969(60.5)	0.304
Age (years)	51.9±26.9	51.7±28.5	51.8±27.6	0.838
Height (cm)	147.6±34.7	152.4±27.8	149.4±32.4	0.025
Weight (kg)	50.4±33.5	49.8±20.6	50.2±29.2	0.717
Systolic blood pressure (mmHg)	118.5±31.5	117.5±20.7	118.1±27.7	0.500
Diastolic blood pressure (mmHg)	68.4±14.4	68.3±14.7	68.3±14.5	0.896
Pulse rate (bpm)	84.5±21.4	85±21.0	84.7±21.2	0.634
BMI ≥25 kg/m2, n (%)	103(16.5)	64(17.3)	167(16.8)	0.725
Diabetes Mellitus, n (%)	340(35.3)	174(27.3)	514(32.1)	0.001
Hypertension, n (%)	409(42.5)	248(38.9)	657(41.0)	0.152
Hyperlipidemia, n (%)	174(18.1)	105(16.5)	279(17.4)	0.405
Chronic Kidney Disease, n (%)	167(17.3)	89(13.9)	256(16.0)	0.070
Cancer, n (%)	440(45.7)	263(41.2)	703(43.9)	0.078
Dep. Internal Medicine, n (%)	215(22.3)	107(16.8)	322(20.1)	0.007
Dep. Surgery, n (%)	285(29.6)	205(32.1)	490(30.6)	0.281
Dep. Minor, n (%)	211(21.9)	155(24.3)	366(22.9)	0.266
Dep. Emergency, n (%)	108(11.2)	58(9.1)	166(10.4)	0.172
Dep. Others, n (%)	144(15.0)	113(17.7)	257(16.1)	0.141
WBC (10^3/μL)	9.1±18.1	9.4±5.4	9.2±14.5	0.717
RBC (10^6/μL)	3.9±0.8	4.0±0.8	4.0±0.8	0.004
Hemoglobin (g/dL)	12.0±2.3	12.1±2.6	12.1±2.4	0.258
Hematocrit (%)	36.5±6.8	36.9±7.4	36.6±7.0	0.259
Platelet (10^3/μL)	229.4±102.3	251.5±115.6	238.2±108.3	<0.001
BUN (mg/dL)	19.8±14.9	18.5±13.2	19.2±14.3	0.083
Creatinine (mg/dL)	1.1±1.5	1.0±1.3	1.1±1.4	0.226
eGFR (mL/min/1.73m2)	69.4±40	68.1±32.7	68.9±37.2	0.540
ASAT (U/L)	24(18,34)	23(18,33)	24(18,34)	0.117
ALT (U/L)	17(11,28)	16(10,25)	17(11,27)	0.480
γGTP (U/L)	29(17,59)	29(19,68)	29(18,61)	0.333
Cholinesterase (U/L)	255.6±101.8	249±83.9	253.4±96.2	0.449
C-reactive protein (mg/dL)	2.4±4.4	3.1±5.3	2.7±4.8	0.006
Total Protein (mg/dL)	6.7±0.9	6.6±0.9	6.7±0.9	0.256
Albumin (mg/dL)	3.6±0.7	3.5±0.7	3.5±0.7	0.408

BMI: body mass index; WBC: white blood cells, RBC: red blood cells, BUN: blood urea nitrogen, eGFR: estimated glomerular filtration rate, ASAT: aspartate aminotransferase, ALT: alanine transaminase, γGTP: gamma-glutamyl transferase
