# Supplementary material for: Antimicrobial resistance patterns of WHO priority pathogens isolated in hospitalized patients in Japan: A tertiary center observational study
Source: PLoS One. 2024 Jan 11;19(1):e0294229. doi: 10.1371/journal.pone.0294229 (PMC10783704; doi:10.1371/journal.pone.0294229)
Supplement: S2 Table — (PDF) [file pone.0294229.s002.pdf]

In-hospital mortality in patients with antimicrobial resistance (AMR) based on World Health Organization priority pathogen list (WHO PPL)

	2010-2015	2016-2021	all	P- value
In-hospital mortality (all priority resistance), n (%)	38/963(3.9)	25/638(3.9)	63/1601(3.9)	0.978
Critical priority resistance, n (%)	1/32(3.1)	4/53(7.5)	5/85(5.9)	0.401
High priority resistance, n (%)	34/805(4.2)	17/499(3.4)	51/1304(3.9)	0.460
Medium priority resistance, n (%)	3/126(2.4)	4/86(4.7)	7/212(3.3)	0.364
